# Deepstacked-AVPs: predicting antiviral peptides using tri-segment evolutionary profile and word embedding based multi-perspective features with deep stacking model

**DOI:** 10.1186/s12859-024-05726-5

**Published:** 2024-03-07

**Authors:** Shahid Akbar, Ali Raza, Quan Zou

**Affiliations:** 1 Institute of Fundamental and Frontier Sciences, University of Electronic Science and Technology of China, Chengdu, 610054 People’s Republic of China; 2 Department of Computer Science, Abdul Wali Khan University Mardan, Mardan, 23200 KP Pakistan; 3 Department of Physical and Numerical Sciences, Qurtuba University of Science and Information Technology, Peshawar, 25124 KP Pakistan; 4 Yangtze Delta Region Institute (Quzhou), University of Electronic Science and Technology of China, Quzhou, 324000 People’s Republic of China

**Keywords:** Antiviral peptides, Prediction, Tri-segmentation based evolutionary features, Word embedding, Feature selection, Stacked ensemble model

## Abstract

**Background:**

Viral infections have been the main health issue in the last decade. Antiviral peptides (AVPs) are a subclass of antimicrobial peptides (AMPs) with substantial potential to protect the human body against various viral diseases. However, there has been significant production of antiviral vaccines and medications. Recently, the development of AVPs as an antiviral agent suggests an effective way to treat virus-affected cells. Recently, the involvement of intelligent machine learning techniques for developing peptide-based therapeutic agents is becoming an increasing interest due to its significant outcomes. The existing wet-laboratory-based drugs are expensive, time-consuming, and cannot effectively perform in screening and predicting the targeted motif of antiviral peptides.

**Methods:**

In this paper, we proposed a novel computational model called Deepstacked-AVPs to discriminate AVPs accurately. The training sequences are numerically encoded using a novel Tri-segmentation-based position-specific scoring matrix (PSSM-TS) and word2vec-based semantic features. Composition/Transition/Distribution-Transition (CTDT) is also employed to represent the physiochemical properties based on structural features. Apart from these, the fused vector is formed using PSSM-TS features, semantic information, and CTDT descriptors to compensate for the limitations of single encoding methods. Information gain (IG) is applied to choose the optimal feature set. The selected features are trained using a stacked-ensemble classifier.

**Results:**

The proposed Deepstacked-AVPs model achieved a predictive accuracy of 96.60%%, an area under the curve (AUC) of 0.98, and a precision-recall (PR) value of 0.97 using training samples. In the case of the independent samples, our model obtained an accuracy of 95.15%, an AUC of 0.97, and a PR value of 0.97.

**Conclusion:**

Our Deepstacked-AVPs model outperformed existing models with a ~ 4% and ~ 2% higher accuracy using training and independent samples, respectively. The reliability and efficacy of the proposed Deepstacked-AVPs model make it a valuable tool for scientists and may perform a beneficial role in pharmaceutical design and research academia.

## Introduction

Viruses are serious and ubiquitous pathogens that cause several high rates of infections and mortality in humans and animals [1]. Viral infections can affect the species for a longer time because of their different variations in transmission, genetic variations, and effective survival in the host cells [2]. Recently, the prevalence of zoonotic viruses such as Zika, Ebola, and the novel SARS-COV-2 causes chronic and killer diseases [3]. Presently, hundreds of different antiviral medications have been developed for treating other families of viruses, i.e., HIV, rhinoviruses, herpes, hepatitis B–C, influenza, etc. [4]. The prevention of viral diseases is challenging owing to inadequate antiviral therapies and a lack of state-of-the-art viral pathogens. Traditional medications suffer from inefficiency, high side effects, and time-consuming procedures [5]. Antiviral peptides (AVPs) are considered one of the key classes of antimicrobial peptides used in developing novel peptide-based powerful therapeutics for treating different viral infections. AVPs are small peptides that can be synthetically obtained using twenty amino acids or chemical clusters into natural peptide samples [6]. AVPs have numerous characteristics, i.e., low side effects, high efficiency, low molecular weight, and low toxicity. It can be widely applied in producing innovative antiviral therapeutics [7].

With huge the growth in genomics data in recent decades, computational intelligence-based data-driven have attained great attention and are considered an alternative for predicting various therapeutic functions in bioinformatics. Consequently, different machine-learning models have been developed for predicting antiviral peptides (AVPs). Initially, Thakur et al. developed the AVPpred model by applying amino acid composition, sequence alignment, physiochemical properties, and motif search for feature formulation [8]. The extracted spaces were trained via the SVM model using a tenfold cross-validation test. AVPpred was trained and validated using two different datasets. Similarly, Chang et al. employed the random forest model by incorporating other sequence encoding methods such as compositional, aggregation, secondary structured, and physiochemical properties [9]. Later on, AVP-IC50Pred applied four different machine learning classifiers using the binary profile, residue composition, and structural features for predicting activity related to AVPs [10]. Furthermore, Nath et al. applied a stacked-ensemble classifier using alignment scoring and an evolutionary descriptors-based feature encoding approach [11]. Lissabet et al. developed the AntiVPP 1.0 predictor for AVPs [12]. The residue composition and relative frequency-based encoding techniques were applied to obtain features from peptide samples. The obtained vector was trained and validated using a random forest (RF) model. Similarly, the PEPred-Suite model utilized adaptive formulation methods for peptide samples for predicting eight different functional types of therapeutic peptides [13]. Whereas the two-level feature selection was employed using the ensemble RF model. Similarly, HybAVPnet presented a two-step training approach for predicting AVPs [14]. The eighteen different encoding techniques were evaluated using light-GBM and neural network models. In the training phase of the HybAVPnet model, the predicted probabilities of the step-1 classifiers were provided to the SVM model for evaluating resultant outcomes. Akbar et al. proposed an ensemble classifier using the transformed-evolutionary and SHAP feature selection-based model for predicting AVPs [15]. Meta-iAVP presented a stacking approach using the predicted scores of SVM, KNN, GLM, RF, regression trees, and XGboost models [16]. Different frequency and amphiphilic-pseudo amino acid compositions were applied for the numerical representation of peptide samples. Pang et al. developed the AVPIden model for predicting the peptide samples with antiviral activities from six different virus families with eight types [17]. Additionally, AVPIden used physiochemical properties, frequency, and gapped-compositional features for peptide representation. Recently, Lin et al. developed AI4AVP for AVPs by training deep convolutional neural networks using a variety of formulation methods [18].

After carefully observing all the above-mentioned studies, we found that each model performs a significant and active role in predicting AVPs. However, these methods are still suffering from reliability and generalization problems. Most existing models applied sequential encoding schemes that only target the residue composition of the individual amino acids without preserving the sequence order information. Some models proposed traditional evolutionary feature descriptors, which are very time-consuming to calculate for each protein sample by searching databases. Additionally, from a training point of view, the existing models were mainly focused on traditional machine learning (ML) based trained models. In contrast, recently, training models via ensemble learning outperformed traditional ML models in bioinformatics. Therefore, we choose a stacked ensemble classifier to effectively train the model using diverse feature representations. The main advantages of stacking over other ensemble classifiers, such as boosting and bagging, include its ability to capture diverse patterns in the input data and leverage the strengths of baseline classifiers. In the case of small training datasets, the stacked ensemble models have shown better performance than boosting and bagging. Moreover, if the relationships between features are complex and cannot easily captured by individual learners, then stacking has an edge over other ensemble classifiers.

**Fig. 1:**
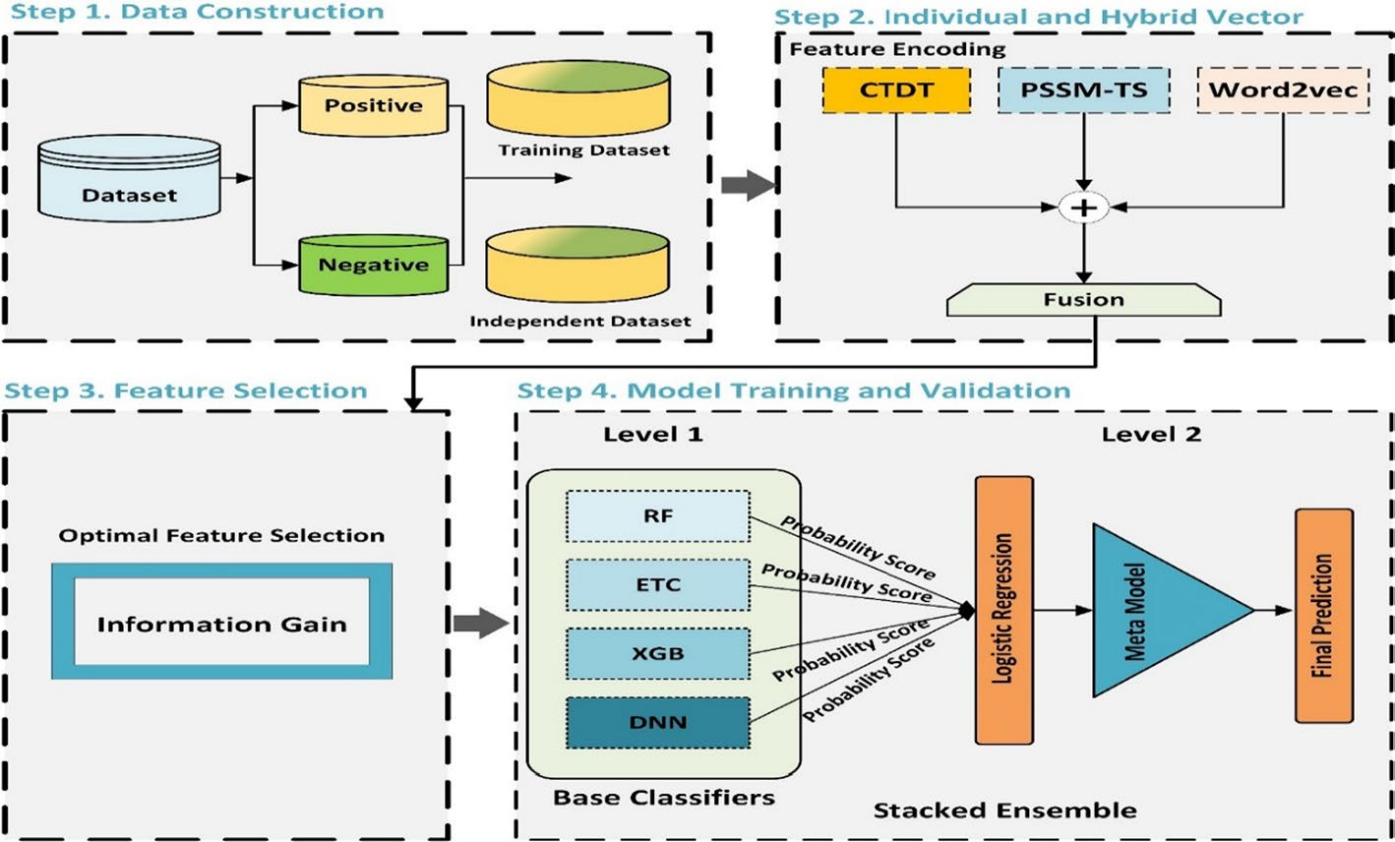
The framework of deepstacked-AVPs prediction model

In this paper, we proposed a stacked-ensemble model, Deepstacked-AVPs, for predicting AVPs. The peptide sequences were numerically formulated using Composition/Transition/Distribution with Transition (CTDT) based physicochemical properties and a word2vec-based skipgram model for capturing semantic information using different k-mer. Apart from these, we developed a novel Position-Specific Scoring Matrix tri-segmentation to form an improved evolutionary matrix named PSSM-TS. A multi-feature is generated using CTDT, K-mer, and PSSM-TS vectors. The Information gain (IG) based feature selection is then employed to gather optimal features for effective prediction of the targeted class. The training process of the proposed model consists of two phases. Initially, four base models, i.e., random forest (RF), extra tree classifier (ETC) XGBoost (XGB), and Deep neural network (DNN), are applied for training individual models. Then, a stacked ensemble-based meta-classifier is formed through logistic regression (LR) using the predicted scores of the individual classifiers [19]. The proposed Deepstacked-AVPs performed remarkably by exhibiting superior performance on both the training and independent datasets. The complete framework of the proposed Deepstacked-AVPs model is depicted in Fig. 1.

## Materials and methods

### Dataset description

In bioinformatics, selecting an appropriate training dataset is crucial in developing an automatic intelligent model [20–22]. The benchmark dataset selection significantly impacts a computational model's performance. The training dataset used in this study was initially constructed in the AVPpred predictor [8]. However, while preparing the dataset, the unnecessary letters such as ‘B’, ‘U’, and ‘X’ were eradicated from the peptide sequences. The used training dataset consists of 951 samples, where 544 are AVPs and 407 are non-AVPs. Moreover, a similar training dataset has been applied for developing various models, such as Chang et al. [9], and AntiVPP 1.0 [12]. Additionally, an independent dataset was used to examine the reliability and generalization of our training model. The independent dataset comprised unseen samples with 60 AVPs and 60 non-AVPs sequences [23]. The selection of independent samples ensured no overlapping between the training and independent datasets to validate the overfitting of our model.

### Feature extraction schemes

#### Position-specific scoring matrix using tri-segmentation (PSSM-TS)

Position-Specific Scoring Matrix (PSSM) can represent the evolutionary profile of the amino acid sequences. However, a simple PSSM vector cannot calculate the sequence ordering information of the local residues [24]. Moreover, the recent computational models observed that local residues of the PSSM descriptor represent the high discriminative and reliable features that lead to achieving high predictive outcomes for different biological problems [25–28]. Hence, we developed a tri-segmentation (TS) concept into a PSSM vector [29]. The PSSM information is divided into three segments by row with equal dimension size. Then, each segment is individually calculated to capture local evolutionary information. At last, all three segments are fused into a single evolutionary vector called PSSM-TS. Each segment Seg-PSSM ($\psi$) can represented as follows:

1 $$Seg{\text{-}}PSSM(\psi ) = [F^{A} ,F^{C} , \ldots ,F^{\psi } ]_{1 \times 20}$$

where $\psi$ signifies the no; of slices and $F^{\psi }$ denotes the residue type of twenty natural amino acid residues in Seg-PSSM. The Tri-segmentation PSSM (TS-PSSM) can be represented as:

2 $$TS{\text{-}}PSSM = [Seg{\text{-}}PSSM(a) + Seg{\text{-}}PSSM(b) + Seg{\text{-}}PSSM(c)].$$

The dimension vector of the proposed TS-PSSM is 60D.

#### Word2Vec-based word embedding

**Fig. 2:**
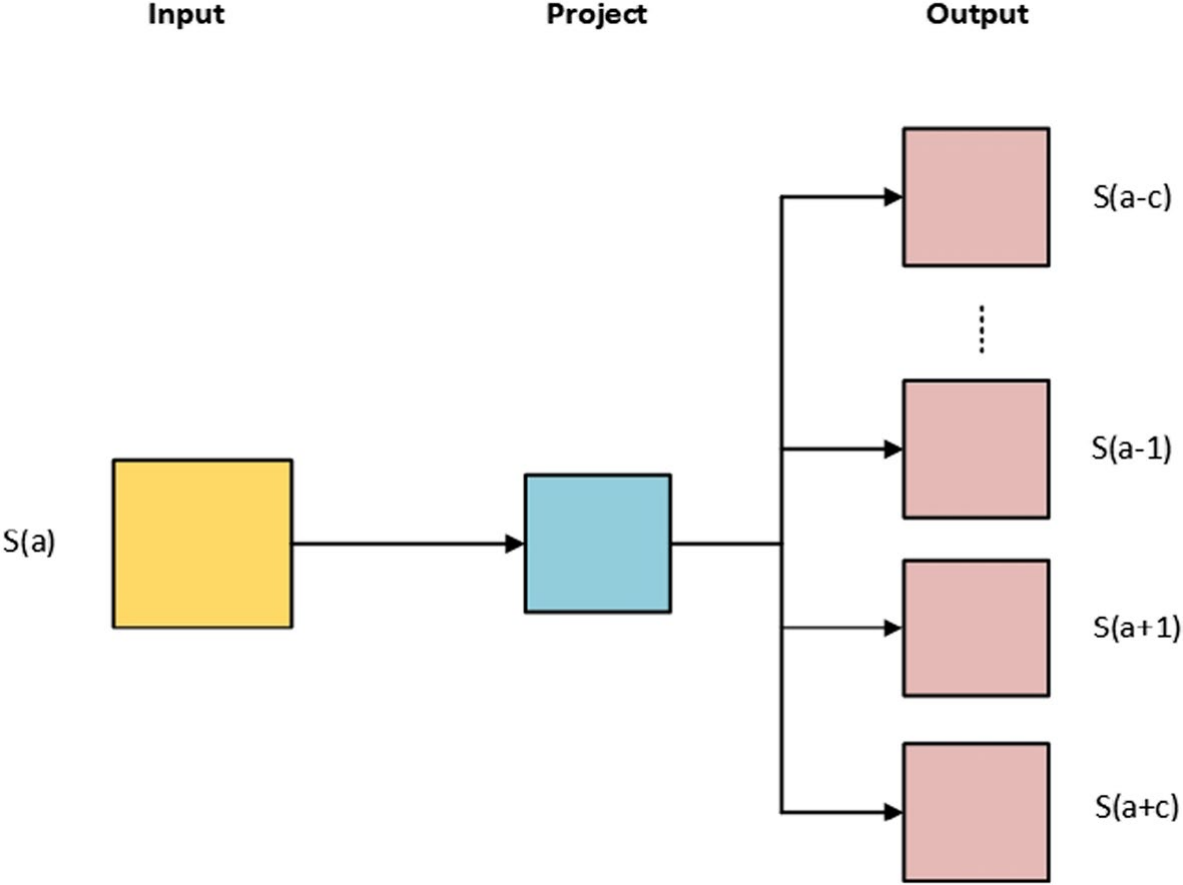
The architecture of the skip-gram model

In the word2vec approach, the contextual relationships among words are captured, yielding distributed representations that encode various linguistic regularities and patterns [30]. Within the protein-encoding process, the segments of k amino acids, commonly referred to as k-mer for treating individual lexical units. Each peptide sequence was segmented into k-mer using the window method, which has been widely employed in natural language processing [31, 32]. In this work, we used a skip-gram model for word representations using different k-mers that are used for the prediction of other words within the peptide sentence. In a given corpus, the skip-gram model is used for training word vectors of each word. For a word $\left( {S\left( a \right)} \right)$ within a sentence, skip-gram can predict the probabilities $P\left( {S\left( {a + i} \right)|S\left( a \right)} \right)$ of the neighboring words $S_{i} \left( {a - k \le i \le a + k} \right)$ depending on the probability of the current word $S\left( a \right)$, as shown in Fig. 2. Whereas, every word space represents the residue of the neighboring words. The key objective of the skip-gram is to maximize the value of E as follows:

3 $$E = \frac{1}{n}\sum\limits_{a = 1}^{n} {\left( {\sum\limits_{ - k \le i \le k,i \ne 0} {log_{2} P\left( {S\left( {a + i} \right)|S\left( a \right)} \right)} } \right)}$$

where the parameter $k$ represents the window size, $S\left( {a + i} \right)$ ($- k \le i \le k$) signifies the K-words neighboring to the current word $S\left( a \right)$, and the term $n$ shows the total number of words.

As mentioned above, that word2vec can capture the residue relationships of the words within the amino acid sample and preserve structural information, we considered the k-mers as “words”. Finally, from each sample, a word embedding vector of 100D is extracted using the skip-gram model.

#### *Composition/transition/distribution with transition (CTDT)*

CTDT is a physiochemical properties-based global distribution method of the peptide sequences [33]. CTDT represents the structural and biochemical characteristics of protein sequences based on different types of groups. The last T in CTDT signifies the transition among three groups of amino acid properties: hydrophobic, polar, and neutral. More specifically, the occurring frequencies of these groups are computed [34, 35]. For two adjacent amino acid residues $\left( {r,s} \right)$, the CTDT features can be calculated as follows:

4 $$CTDT\left( {r,s} \right) = \frac{{N\left( {r,s} \right) + N\left( {s,r} \right)}}{N}$$

where the residue pairs $\left( {r,s} \right) \in \left\{ {\left( {positive, \, neutral} \right), \, \left( {neutral, \, negative} \right), \, \left( {negative, \, positive} \right)} \right\}$, $N\left( {r,s} \right)$ and $N\left( {s,r} \right)$ represents the frequency counts composed of “$r,s$” and “$s,r$” within the protein peptide sequence. The analysis was performed using thirteen distinct physicochemical properties. The resultant CTDT feature vector comprises 39 features against each sample.

### Information gain

In the feature engineering phase, redundant features can affect the predictive accuracy of a training model [36]. Therefore, feature selection techniques are commonly employed to identify and choose the most relevant features from the extracted space for improving the classification rates with minimum computational cost [37, 38]. In this work, we used information gain (IG) as a feature selection for identifying the most significant features by examining the reduction in entropy by splitting training samples based on a value of a random attribute [39]. The high IG value represents the low entropy.

For given training samples denoted by “$S$” with attributes represented by $A$, the IG ($S$,$A$) associated with the attribute $A$ can be defined by reducing the entropy observed within the training samples when the attribute $A$ is considered [40]. Which can be expressed mathematically represented as follows:

5 $$IG\left( {S,A} \right) = H\left( S \right) - H\left( {{S \mathord{\left/ {\vphantom {S A}} \right. \kern-0pt} A}} \right)$$

$H\left( S \right)$ represents the entropy of the training samples $S$, and $H\left( {{S \mathord{\left/ {\vphantom {S A}} \right. \kern-0pt} A}} \right)$ signifies the entropy of the training samples $S$ under the condition that the attribute $A$ has been observed. Which is particularly relevant in the classical scenario of a dichotomous classification:

6 $$H\left( S \right) = - \sum\limits_{l = 1}^{2} {p_{l} log_{2} p_{l} }$$

and

7 $$H\left( {{S \mathord{\left/ {\vphantom {S A}} \right. \kern-0pt} A}} \right) = \sum\limits_{v \in Values\left( A \right)} {\frac{{|S_{v} |}}{|S|}H\left( {S_{v} } \right)}$$

here the notation $Values\left( A \right)$ denotes the collection of all possible values associated with the attribute $A$. Additionally, $S_{v}$ stands for the partition of the training dataset that corresponds to the specific value “*V*” of the attribute $A$. The entropy of this partition is calculated by $H\left( {S_{v} } \right)$. The vertical bars (|.|) represent the cardinality operator [40].

In this work, 89 optimal features were selected using IG-ranking-based feature selection. Where the ranking of the features was established in descending order. This ranking assigns the highest priority to the most substantial Information gain attribute.

### UMAP-based features visualization

To demonstrate the effectiveness of the extracted features of our proposed model, we employed a Uniform Manifold Approximation and Projection (UMAP) based statistical visualization technique [41].UMAP is a data visualization approach that is used to preserve not only local structural information but also global structural relationships. In data visualization, the samples of two classes as represented as two different clusters. Which is useful for comprehending the relationship among samples of different classes used for discrimination of peptides. In this study, we performed the UMAP visualization of the extracted features of the training samples i.e., CTDT, Word2Vec, PSSM-TS, Hybrid features, IG-based optimal features, and independent samples as shown in Fig. 5.

### Model architecture of deepstacked-AVPs model

The Stacking model used in this study mainly comprises two phases [42]. Initially, the baseline models, namely ETC [43], RF [44], XGB [28], and DNN [45], are trained based on the extracted vectors from the training dataset. The grid search approach is employed to select the optimal model training parameters. In the case of the DNN model, two dense layers were added after the input layer to facilitate feature matrix extraction. The Rectified Linear Unit (ReLU) activation function is also applied to deal with nonlinearity in the two dense layers. The Adam and early stop and dropout strategies are also used to reduce the risk of overfitting.

In the second phase, a meta-classifier is formed using logistic regression (LR) by computing the predicted probability scores of the baseline classifiers. The probability scores are in the range of (0–1). Where a threshold of 0.5 is used to define the predicted class of a protein sample, i.e., the probability > 0.5 will predict label 1, and < 0.5 will predict label 0 class. Implementing the LR-based stacked-ensemble classifier has significantly increased predictive rates compared to individual baseline models.

### Experimental configuration

Our proposed model is established through the utilization of Intel (R) Xeon(R) @ 3.3 GHz, providing a RAM of 64 GB. For code implementation, we utilized the Windows 10 operating system and Python 3.10.6 as the programming language. Additionally, several Python libraries were used in the model training process.

## Performance evaluation

In bioinformatics and applied machine learning, various performance parameters are utilized to assess the predictive capabilities of the training models [46, 47]. Mostly, in binary class problems, a confusion matrix is formed to store the prediction outcomes of the training models, i.e., True Positives (TP), True Negatives (TN), False Positives (FP), and False Negatives (FN). The predictive accuracy is often the primary metric for model effectiveness assessment [48, 49]. However, to comprehensively evaluate a model, we employed the following performance evaluation metrics to assess our model more rigorously.

8 $$Acc = \frac{TP + TN}{{\left( {TP + TN + FP + FN} \right)}}$$

9 $$Sn = \frac{TP}{{\left( {TP + FN} \right)}}$$

10 $$Sp = \frac{TN}{{\left( {TN + FP} \right)}}$$

11 $$MCC = \frac{TP \times TN - FP \times FN}{{\sqrt {\left( {TP + FP} \right)\left( {TP + FN} \right)\left( {TN + FP} \right)\left( {TN + FN} \right)} }}$$

where Acc, Sn, Sp, and MCC represent the accuracy, sensitivity, specificity, and Matthew’s coefficient correlation, respectively.

## Results and discussion

In this study, we evaluated our model using a fivefold cross-validation test, where data in each fold was randomly selected [50]. Additionally, to achieve reliable results from the random distribution of data among the folds, we selected the mean value of the fold-CV test by repeating the stratified loop procedure 50 times [50, 51]. In the below subsections, we will thoroughly discuss the classification outcomes of the individual and ensemble training models before and after applying feature selection.

### Prediction analysis of classification models using training samples

The prediction results of the individual feature vectors using training peptides are given in Table 2. We mentioned above that we employed three different extraction methods, CTDT, word2vec, and PSSM-TS to numerically transform the peptide samples. At first, the extracted vectors were evaluated individually using four machine-learning models: RF, ETC, XGB, and DNN. Whereas, the optimal parameters used for training the individual machine-learning models are provided in Table 1. In the case of CTDT features using individual classifiers, RF and DNN obtained better accuracies of 88.07% and 88.01% than ETC and XGB, respectively. While the stacked-ensemble model achieved an accuracy of 89.90% with Sn of 93.93%, Sp of 86.55%, MCC of 0.80, and AUC of 0.95. Evaluating the individual classifiers using the evolutionary vector of PSSM-TS, the ETC model reported a predictive accuracy of 87.61%, Sp of 90.91%, and AUC of 0.94. While PSSM-TS features using a stacked-ensemble model obtained an accuracy of 89.10% with an AUC of 0.96. On the other hand, 3-mer of the skip-gram model using RF achieved an accuracy of 88.10%, with Sn of 91.88%, and AUC of 0.92. While the ensemble stacked learner using 3-mer reported an 89.91%, with sp of 90.23%, and AUC of 0.96. Finally, the hybrid vector (CTDT + PSSM-TS + 3mers) using the stacking model obtained an improved accuracy of 92.20%, Sn of 90.75%, Sp of 93.91%, MCC of 0.84, and AUC of 0.96. In comparison with all training models, the Stacked-Ensemble model consistently achieved the highest predictive results, demonstrating its exceptional discriminative power.

**Table 1:** Hyper parameters of classifiers learning model

Methods	Parameter	Optimal value
DNN	Activation function	ReLu, sigmoid
	Learning rate	0.01
	Number of hidden layer Neurons	64,32,16
	Optimizer	Adam
	Regularization L1	0.001
	Dense layers	3
	Dropout	0.25,0.5
RF	n_estimators	200
	Random_state	42
	Max features	Auto
	Max_depth	32
	Bootstrap	TRUE
	min_samples_leaf	4
	min_samples_split	10
XGB	n_estimators	200
	LEARNING rate	0.001
	Max depth	15
	reg_lambda	2
	Objective function	Binary-logistic
	Gamma	1
	Booster	Gbtree
	reg_alpha	1
ETC	Random_state	42
	n_estimators	150
	Criterion	Entropy
	Max_features	Sqrt

### Prediction analysis of classifiers using information gain based feature selection

**Fig. 3:**
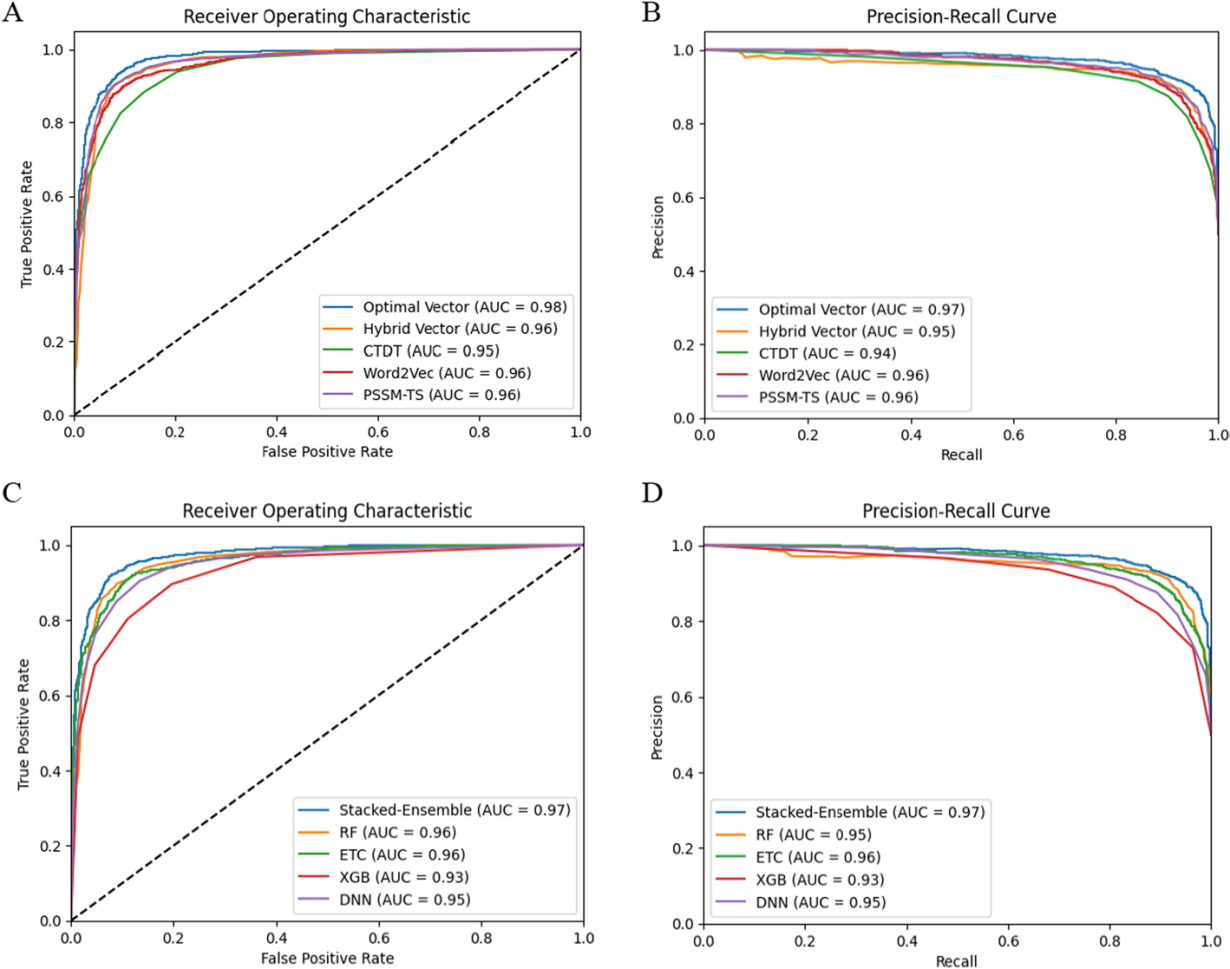
**A** ROC analysis of Training samples, **B** PR analysis of Training samples, **C** ROC analysis of independent samples, **D** PR analysis of independent samples

**Fig. 4:**
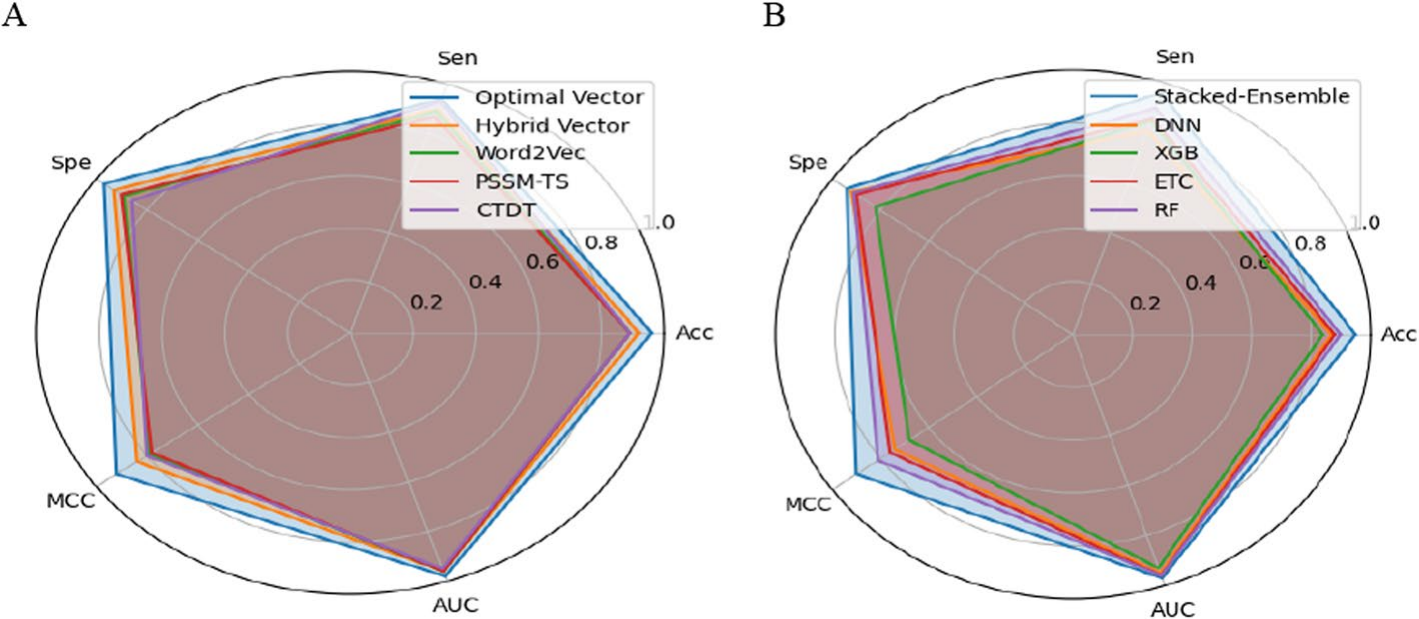
Comparison of individual, hybrid, and optimal vectors using **A** Training samples, **B** Independent samples

In bioinformatics-based machine learning models, feature selection performs a key role by proposing a cost-effective computational model. Selecting highly relevant features from the training vector improves performance [52, 53]. As described in Table 2, the hybrid vector achieved higher results than the individual vectors. Hence, we applied information gain (IG) for selecting highly relevant features from the hybrid vector. The hybrid vector (CTDT + 3-mer + PSSM-TS) contains 199 features with 39 features of CTDT, 100 features of 3-mer, and 60 features were obtained using PSSM-TS. After employing IG feature selection, only 50 optimal features were selected from the whole hybrid vector. Like the training samples, the selected feature vector was also evaluated using RF, ETC, XGB, and DNN classifiers. The evaluation results of the chosen vector are provided in Table 3, which have effectively shown their contribution by accurately discriminating the targeted classes. In the case of individual learning models using selected features, the ETC, XGB, and DNN achieved accuracies of 89.44%, 89.16%, and 89.90%. In comparison, the RF training model performed well by reporting an accuracy of 91.28% and an AUC of 0.96. Furthermore, the Stacked-Ensemble model boosted the results by obtaining an accuracy of 96.60%, with sensitivity, specificity, and MCC values of 94.85%, 97.35%, and 0.92, respectively. The performance of the individual features, hybrid features, and optimal features using training samples are compared in Fig. 4. The higher predictive results show the effectiveness of selecting highly discriminative features via IG feature selection. Moreover, the proposed method is validated using an independent dataset to show its generalization power as provided in Table 4. The instance-based AUC and precision-recall (PR) analysis of the training and independent features are provided in Fig. 3.

**Table 2:** Predictive outcomes of the training dataset via different feature descriptors

Encoding method	Classifier	Acc (%)	Sn (%)	Sp (%)	MCC	AUC
CTDT	RF	88.07	90.90	85.71	0.76	0.95
	ETC	72.47	79.79	66.38	0.46	0.80
	XGB	82.11	78.78	84.87	0.63	0.90
	DNN	88.01	83.19	88.89	0.76	0.93
	Stacked-ensemble	89.90	93.93	86.55	0.80	0.95
PSSM-TS	RF	87.15	83.19	91.91	0.74	0.93
	ETC	87.61	84.87	90.91	0.75	0.94
	XGB	77.06	83.19	69.69	0.53	0.84
	DNN	86.23	89.07	82.82	0.72	0.91
	Stacked-ensemble	89.10	87.39	90.90	0.78	0.96
Word2vec (3mer)	RF	88.10	84.87	91.88	0.76	0.92
	ETC	74.31	78.99	68.61	0.47	0.76
	XGB	78.44	76.47	80.81	0.57	0.85
	DNN	87.61	84.48	91.90	0.76	0.92
	Stacked-ensemble	89.91	89.97	90.23	0.79	0.96
Hybrid vector	RF	88.91	85.71	92.92	0.78	0.94
	ETC	88.07	83.19	93.93	0.76	0.94
	XGB	87.61	88.23	86.85	0.75	0.93
	DNN	88.53	93.93	84.03	0.77	0.95
	Stacked-ensemble	92.20	90.75	93.91	0.84	0.96

**Table 3:** Predictive outcomes of the hybrid vector of training samples after applying information gain

Dataset	Classifier	Acc (%)	Sn (%)	Sp (%)	MCC	AUC
Training dataset	RF	91.28	90.75	91.90	0.82	0.96
	ETC	89.44	89.91	88.81	0.78	0.95
	XGB	89.16	88.33	90.10	0.78	0.96
	DNN	89.90	88.23	91.91	0.79	0.95
	Stacked-ensemble	96.60	94.85	97.35	0.92	0.98

**Table 4:** Predictive outcomes of deepstacked-AVPs model using independent dataset

Classifier	Acc (%)	Sn (%)	Sp (%)	MCC	AUC
RF	90.83	90.00	91.66	0.81	0.96
ETC	88.33	86.66	90.00	0.76	0.96
XGB	84.16	85.81	82.50	0.68	0.93
DNN	87.50	81.80	92.30	0.74	0.95
Stacked-ensemble	95.15	96.27	94.91	0.90	0.97

### Comparison of deepstacked-AVPs model with existing predictors

**Fig. 5:**
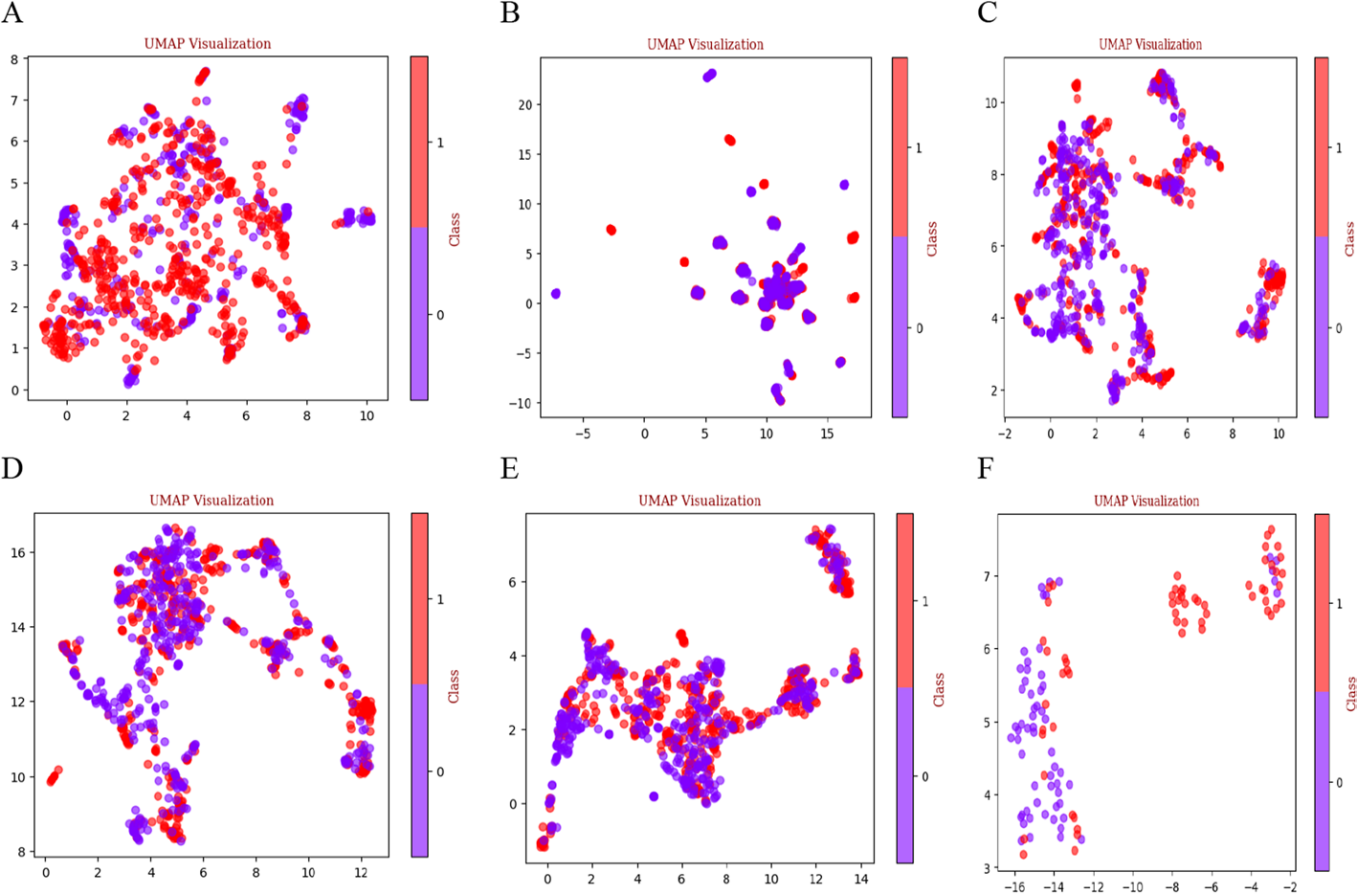
UMAP visualization of training samples using **A** CTDT, **B** Word2Vec, **C** PSSM-TS, **D** Hybrid features, **E** IG-based optimal features and **F** independent samples

To assess the efficacy of our study, we conducted a comparative analysis of our predictor with existing models using training and independent datasets as shown in Table 5. In the case of training samples, the AVPpred model using sequential and physiochemical properties based motif descriptors ACC of 85%, with Sn, Sp, and MCC of 82.20%, 88.20%, and 0.70, respectively [8]. Similarly, Chang et al. trained the RF model using the same training samples using compositional residue encoding, aggregation, and secondary structured features [9]. Their model achieved an ACC of 85.10%, Sn of 86.60%, Sp of 83%, and MCC of 0.70. Meta-iAVP used physiochemical properties based on computational features by applying the stacking concept [16]. The predicted probabilities of six machine learning models were provided to the stacking algorithm, and obtained an ACC of 88.20%, Sn, Sp, and MCC of 89.20%, 86.90%, and 0.76, respectively. Further, the FIRM-AVP predictor obtained the optimal ranking based on numerical features using the structural and physicochemical properties of peptides [54]. FIRM-AVP reported an ACC of 92.40%, Sn of 93.30%, Sp of 91.10%, and MCC of 0.84. In contrast, our proposed Deepstacked-AVPs model outperformed by improving the predictive rates of 4.2%, 6.25%, and 0.08 higher ACC, Sp, and MCC, respectively. Apart from these, using an independent dataset, the AVPpred model obtained 92.50% ACC, with 93.30% sn, 91.70% sp, and MCC of 0.85 [8]. Chang et al. reported an ACC of 93.30%, and sp of 95% [9]. Likewise, Meta-iAVP achieved an ACC of 94.90%, Sp of 98.30%, and MCC of 0.90 [16]. Furthermore, AntiVPP 1.0 using independent samples achieved an ACC of 93%, Sp of 97%, and MCC of 0.87 [12]. While our proposed Deepstacked-AVPs model surpassed the existing model, by achieving higher prediction outcomes with 2.17% ACC, 9.27% Sn, 2.91% Sp, and 0.03 improvement in MCC value (Figs. 4, 5).

**Table 5:** Performance comparison of deepstacked-AVPs method with existing models

Dataset	Predictor	Acc (%)	Sn (%)	Sp (%)	MCC
Training dataset	AVPpred [8]	85.00	82.20	88.20	0.70
	Chang et al. [9]	85.10	86.60	83.00	0.70
	Meta-iAVP [16]	88.20	89.20	86.90	0.76
	Chowdhury et al. [54]	92.40	93.30	91.10	0.84
	Deepstacked-AVPs	96.60	94.85	97.35	0.92
Independent dataset	AVPpred [8]	92.50	93.30	91.70	0.85
	Chang et al. [9]	93.30	91.70	95.00	0.87
	Meta-iAVP [16]	94.90	91.70	98.30	0.90
	AntiVPP 1.0 [12]	93.00	87.00	97.00	0.87
	Deepstacked-AVPs	95.15	96.27	94.91	0.90

## Discussion

**Fig. 6:**
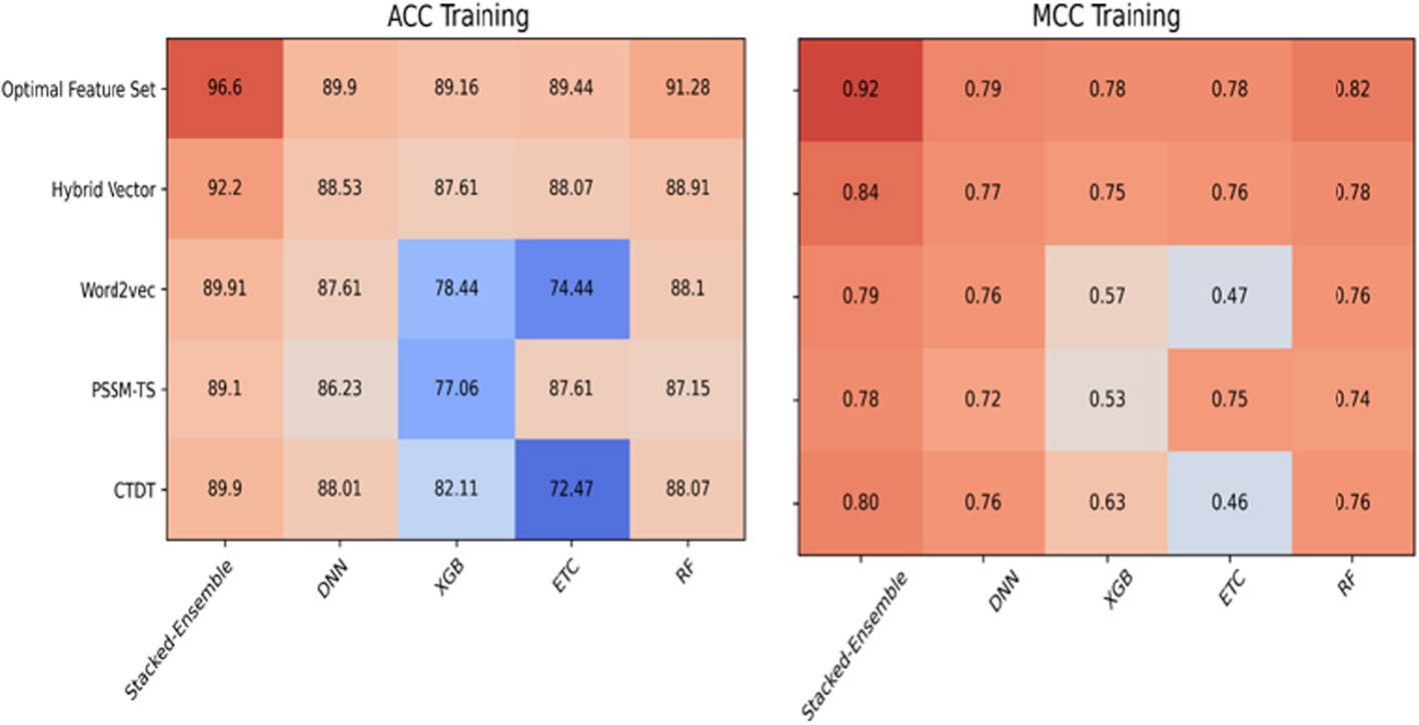
Performance analysis of different encoding methods using baseline classifiers

AVPs are one major class of antimicrobial peptides used for developing novel peptide-based therapeutics to treat various viral infections. Existing traditional laboratory-based methods are laborious and inefficient due to their limited reliability. In this study, we introduce a stacked ensemble model namely, Deepstacked-AVPs, for the accurate discrimination of AVPs and non-AVPs. Initially, four different baseline models were trained using PSSM-TS-based improved evolutionary features, CTDT-based physiochemical properties, and word2Vec-based features. The predicted probability scores of the baseline models are provided for logistic regression to form a stacked ensemble model. To further investigate the extracted features, the hybrid vector is examined using the stacking model, resulting in an ACC of 92.20%, Sp of 93.91%, and AUC of 0.96. The hybrid vector has shown substantial improvement in terms of all evaluation parameters by compensating for the weakness of the individual vectors as shown in Fig. 6. However, to develop a fast training model with minimal computational cost, IG is applied to choose 89 optimal features. The optimal feature set has shown further improvement by achieving an ACC of 96.60%, Sp of 97.35%, and AUC of 0.98. Our proposed model, using training samples, reported 4% higher ACC, 6% higher Sp, and 8% higher MCC than existing state-of-the-art predictors, as provided in Table 5. The generalization and overfitting of the Deepstacked-AVPs model is validated using independent samples and reported improved ACC, Sn, Sp, and MCC of ~ 2%, 9%, 2%, and 3%, respectively. Hence, representing the peptide samples using a multi-informative vector, specifically formulating segmented local features using the novel PSSM-TS approach, and leveraging the powerful training abilities of the stacked-ensemble model significantly improved the performance. Hence, the improved predictive outcomes of the stacking training model are due to the diversity of applied baseline classifiers. Moreover, the stacking model provides flexibility in selecting baseline models, and its generalization capabilities help in mitigating model overfitting, leading to producing robust and reliable predictions. However, the stacking model still suffers from several issues such as the risk of model dependency, computational cost, and hyperparameter tuning. In the future, we will focus on handling these issues.

## Conclusion

In this paper, we developed a Deepstacked-AVPs model to predict antiviral peptides effectively. Keeping the limitations in the existing feature formulation techniques, we numerically represented the amino acid samples using word2vec-based word embedding, PSSM-TS-based improved evolutionary features, and CTDT-based physiochemical properties methods. A multi-informative vector is formed by fusing Word2vec, PSSM-TS, and CTDT vectors. An information gain scheme is applied to develop a computationally-effective model by choosing the optimal feature space from the hybrid vector. Subsequently, the Deepstacked-AVPs meta-model was trained using the probability scores of the individual classifiers. The Deepstacked-AVPs model exhibits consistency and stability by achieving superior accuracy of 96.60% on the training samples and 95.15% on the independent dataset. Our model has outperformed state-of-the-art methods and will significantly contribute to antiviral peptides-related drug design and the pharmaceutical industry.

## Data Availability

The data and source code are available in the public repository: https://github.com/Intelligent-models/Deepstacked-AVPs.

## References

[1] Sébastien Calvignac-Spencer AD, Gogarten JF, Leendertz FH, Patrono LV (2021). Chapter one—a great ape perspective on the origins and evolution of human viruses. Adv Virus Res.

[2] Md Mamunul Islam DK (2022). Toward a next-generation diagnostic tool: a review on emerging isothermal nucleic acid amplification techniques for the detection of SARS-CoV-2 and other infectious viruses. Analytica Chimica Acta.

[3] Phan T (2020). Genetic diversity and evolution of SARS-CoV-2. Infect Genet Evol.

[4] Erik De Clercq GL (2016). Approved antiviral drugs over the past 50 years. Clin Microbiol Rev.

[5] Axel Hollmann NPC, Espeche JC, Maffí PC (2021). Review of antiviral peptides for use against zoonotic and selected non-zoonotic viruses. Peptides.

[6] Iris Oz Gleenberg AH, Hizi A (2007). Inhibition of the activities of reverse transcriptase and integrase of human immunodeficiency virus type-1 by peptides derived from the homologous viral protein R (Vpr). J Mol Biol.

[7] Ke Yan HL, Guo Y, Chen Y, Wu H, Liu B (2022). TPpred-ATMV: therapeutic peptide prediction by adaptive multi-view tensor learning model. Bioinformatics.

[8] Thakur N, Qureshi A, Kumar M (2012). AVPpred: collection and prediction of highly effective antiviral peptides. Nucleic Acids Res.

[9] Chang KY, Yang J-R (2013). Analysis and prediction of highly effective antiviral peptides based on random forests. PLoS ONE.

[10] Qureshi A, Tandon H, Kumar M (2015). AVP-IC50Pred: multiple machine learning techniques-based prediction of peptide antiviral activity in terms of half maximal inhibitory concentration (IC50). Pept Sci.

[11] Nath A (2021). Prediction for understanding the effectiveness of antiviral peptides. Comput Biol Chem.

[12] Lissabet JFB, Belén LH, Farias JG (2019). AntiVPP 1.0: a portable tool for prediction of antiviral peptides. Comput Biol Med.

[13] Wei L, Zhou C, Su R, Zou Q (2019). PEPred-Suite: improved and robust prediction of therapeutic peptides using adaptive feature representation learning. Bioinformatics.

[14] Ge R, Xia Y, Jiang M, Jia G, Jing X, Li Y, Cai Y. HybAVPnet: a novel hybrid network architecture for antiviral peptides identification. bioRxiv 2022:2022.2006. 2010.495721

[15] Akbar S, Ali F, Hayat M, Ahmad A, Khan S, Gul S (2022). Prediction of antiviral peptides using transform evolutionary & SHAP analysis based descriptors by incorporation with ensemble learning strategy. Chemom Intell Lab Syst.

[16] Schaduangrat N, Nantasenamat C, Prachayasittikul V, Shoombuatong W (2019). Meta-iAVP: a sequence-based meta-predictor for improving the prediction of antiviral peptides using effective feature representation. Int J Mol Sci.

[17] Pang Y, Yao L, Jhong J-H, Wang Z, Lee T-Y (2021). AVPIden: a new scheme for identification and functional prediction of antiviral peptides based on machine learning approaches. Brief Bioinform.

[18] Lin T-T, Sun Y-Y, Wang C-T, Cheng W-C, Lu I-H, Lin C-Y, Chen S-H (2022). AI4AVP: an antiviral peptides predictor in deep learning approach with generative adversarial network data augmentation. Bioinform Adv.

[19] LaValley MP (2008). Logistic regression. Circulation.

[20] Feng P, Chen W, Lin H (2016). Identifying antioxidant proteins by using optimal dipeptide compositions. Interdiscip Sci Comput Life Sci.

[21] Meng C, Jin S, Wang L, Guo F, Zou Q (2019). AOPs-SVM: a sequence-based classifier of antioxidant proteins using a support vector machine. Front Bioeng Biotechnol.

[22] Ahmed S, Arif M, Kabir M, Khan K, Khan YD (2022). PredAoDP: accurate identification of antioxidant proteins by fusing different descriptors based on evolutionary information with support vector machine. Chemom Intell Lab Syst.

[23] Zhang L, Zhang C, Gao R, Yang R, Song Q (2016). Sequence based prediction of antioxidant proteins using a classifier selection strategy. PLoS ONE.

[24] Barukab O, Ali F, Alghamdi W, Bassam Y, Khan SA (2022). DBP-CNN: deep learning-based prediction of DNA-binding proteins by coupling discrete cosine transform with two-dimensional convolutional neural network. Expert Syst Appl.

[25] Ali F, Akbar S, Ghulam A, Maher ZA, Unar A, Talpur DB (2021). AFP-CMBPred: computational identification of antifreeze proteins by extending consensus sequences into multi-blocks evolutionary information. Comput Biol Med.

[26] Akbar S, Khan S, Ali F, Hayat M, Qasim M, Gul S (2020). iHBP-DeepPSSM: identifying hormone binding proteins using PsePSSM based evolutionary features and deep learning approach. Chemom Intell Lab Syst.

[27] Akbar S, Mohamed HG, Ali H, Saeed A, Ahmed A, Gul S, Ahmad A, Ali F, Ghadi YY, Assam M. Identifying neuropeptides via evolutionary and sequential based multi-perspective descriptors by incorporation with ensemble classification strategy. IEEE Access 2023.

[28] Akbar S, Ali H, Ahmad A, Sarker MR, Saeed A, Salwana E, Gul S, Khan A, Ali F. Prediction of amyloid proteins using embedded evolutionary & ensemble feature selection based descriptors with eXtreme gradient boosting model. IEEE Access 2023.

[29] Khan A, Uddin J, Ali F, Kumar H, Alghamdi W, Ahmad A (2023). AFP-SPTS: an accurate prediction of antifreeze proteins using sequential and pseudo-tri-slicing evolutionary features with an extremely randomized tree. J Chem Inf Model.

[30] Mikolov T, Sutskever I, Chen K, Corrado GS, Dean J. Distributed representations of words and phrases and their compositionality. Adv Neural Inf Process Syst 2013;26.

[31] Compeau PE, Pevzner PA, Tesler G (2011). How to apply de Bruijn graphs to genome assembly. Nat Biotechnol.

[32] Aggarwala V, Voight BF (2016). An expanded sequence context model broadly explains variability in polymorphism levels across the human genome. Nat Genet.

[33] Govindan G, Nair AS. Composition, transition and distribution (CTD)—a dynamic feature for predictions based on hierarchical structure of cellular sorting. In: 2011 annual IEEE India conference: 2011. IEEE. pp. 1–6.

[34] Chen Z, Zhao P, Li F, Leier A, Marquez-Lago TT, Wang Y, Webb GI, Smith AI, Daly RJ, Chou K-C (2018). iFeature: a python package and web server for features extraction and selection from protein and peptide sequences. Bioinformatics.

[35] Li F, Guo X, Xiang D, Pitt ME, Bainomugisa A, Coin LJ (2022). Computational analysis and prediction of PE_PGRS proteins using machine learning. Comput Struct Biotechnol J.

[36] Ding C, Peng H (2005). Minimum redundancy feature selection from microarray gene expression data. J Bioinform Comput Biol.

[37] Koller D, Sahami M. Toward optimal feature selection. In: ICML: 1996, vol. 292.

[38] Langley P (1994). Selection of relevant features in machine learning: Defense Technical Information Center.

[39] Kandaswamy KK, Pugalenthi G, Hartmann E, Kalies K-U, Möller S, Suganthan P, Martinetz T (2010). SPRED: a machine learning approach for the identification of classical and non-classical secretory proteins in mammalian genomes. Biochem Biophys Res Commun.

[40] Mitchell TM. Machine learning; 1997.

[41] Jinyue Wang SZ, Qiao H, Wang J (2021). UMAP-DBP: an improved DNA-binding proteins prediction method based on uniform manifold approximation and projection. Protein J.

[42] Ahmad S, Charoenkwan P, Quinn JM, Moni MA, Hasan MM, Lio P, Shoombuatong W (2022). SCORPION is a stacking-based ensemble learning framework for accurate prediction of phage virion proteins. Sci Rep.

[43] Peng L, Yuan R, Shen L, Gao P, Zhou L (2021). LPI-EnEDT: an ensemble framework with extra tree and decision tree classifiers for imbalanced lncRNA-protein interaction data classification. BioData Min.

[44] Ao C, Zhou W, Gao L, Dong B, Yu L (2020). Prediction of antioxidant proteins using hybrid feature representation method and random forest. Genomics.

[45] Akbar S, Hayat M, Tahir M, Khan S, Alarfaj FK (2022). cACP-DeepGram: classification of anticancer peptides via deep neural network and skip-gram-based word embedding model. Artif Intell Med.

[46] Dwivedi AK (2018). Performance evaluation of different machine learning techniques for prediction of heart disease. Neural Comput Appl.

[47] Baratloo A, Hosseini M, Negida A, El Ashal G. Part 1: simple definition and calculation of accuracy, sensitivity and specificity; 2015. PMC4614595 26495380

[48] Raza A, Uddin J, Almuhaimeed A, Akbar S, Zou Q, Ahmad A (2023). AIPs-SnTCN: predicting anti-inflammatory peptides using fastText and transformer encoder-based hybrid word embedding with self-normalized temporal convolutional networks. J Chem Inf Model.

[49] Akbar S, Raza A, Al Shloul T, Ahmad A, Saeed A, Ghadi YY, Mamyrbayev O, Eldin ET. pAtbP-EnC: identifying anti-tubercular peptides using multi-feature representation and genetic algorithm based deep ensemble model. IEEE Access 2023.

[50] Akbar S, Hayat M (2018). iMethyl-STTNC: Identification of N6-methyladenosine sites by extending the idea of SAAC into Chou's PseAAC to formulate RNA sequences. J Theor Biol.

[51] Ahmad A, Akbar S, Tahir M, Hayat M, Ali F (2022). iAFPs-EnC-GA: identifying antifungal peptides using sequential and evolutionary descriptors based multi-information fusion and ensemble learning approach. Chemom Intell Lab Syst.

[52] Ali F, Ahmed S, Swati ZNK, Akbar S (2019). DP-BINDER: machine learning model for prediction of DNA-binding proteins by fusing evolutionary and physicochemical information. J Comput Aided Mol Des.

[53] Sikander R, Ghulam A, Ali F (2022). XGB-DrugPred: computational prediction of druggable proteins using eXtreme gradient boosting and optimized features set. Sci Rep.

[54] Chowdhury AS, Reehl SM, Kehn-Hall K, Bishop B (2020). Webb-Robertson B-JM: Better understanding and prediction of antiviral peptides through primary and secondary structure feature importance. Sci Rep.

